# Protons in Gating the Kv1.2 Channel: A Calculated Set of Protonation States in Response to Polarization/Depolarization of the Channel, with the Complete Proposed Proton Path from Voltage Sensing Domain to Gate

**DOI:** 10.3390/membranes12070718

**Published:** 2022-07-20

**Authors:** Alisher M. Kariev, Michael E. Green

**Affiliations:** Department of Chemistry and Biochemistry, The City College of the City University of New York, New York, NY 10031, USA; akariev@ccny.cuny.edu

**Keywords:** ion channel gating, proton transport paths, amino acid strings

## Abstract

We have in the past proposed that proton motion constitutes the gating current in the potassium channel K_v_1.2 and is responsible for the gating mechanism. For this to happen, there must be a proton path between the voltage-sensing domain (VSD) and the channel gate, and here we present quantum calculations that lead to a specific pair of proton paths, defined at the molecular level, with well-defined water molecule linkages, and with hydrogen bonding between residues; there is also at least one interpath crossover, where protons can switch paths. Quantum calculations on the entire 563-atom system give the complete geometry, the energy, and atomic charges. Calculations show that three specific residues (in the pdb 3Lut numbering, H418, E327, R326), and the T1 intracellular moiety, all of which have been shown experimentally to be involved in gating, would necessarily be protonated or deprotonated in the path between the VSD and the gate. Hydroxyl reorientation of serine and threonine residues are shown to provide a means of adjusting proton directions of motion. In the deprotonated state for K312, a low energy state, our calculations come close to reproducing the X-ray structure. The demonstration of the existence of a double proton path between VSD and gate supports the proposed proton gating mechanism; when combined with our earlier demonstration of proton generation in the VSD, and comparison with other systems that are known to move protons, we are close to achieving the definition of a complete gating mechanism in molecular detail. The coupling of the paths to the VSD, and to the PVPV section that essentially forms the gate, can be easily seen from the results of the calculation. The gate itself remains for further computations.

## 1. Introduction

Voltage-gated potassium channels of the type we are discussing are found in practically all forms of life, and in all parts of eukaryotic organisms, with a wide variety of physiological roles. Given their importance, there has long been a huge amount of research interest in them, and considerable structural and functional information has been determined [1]. They have four voltage-sensing domains (VSD), each with four transmembrane (TM) segments; one of these, labeled S4, contains several arginine amino acids that are generally presumed to be always positively charged, while there are counter charges on other TM segments, forming salt bridges [2]. In addition, there are two TM segments from each of the four domains that together form a central pore, through which ions pass when a gate opens; when the membrane is polarized, the gate is closed, so no ions pass. A transient capacitative current accompanies gating (opening) of these and of sodium channels [3,4], so there is no question that charges are moving as the gate opens to allow ions to pass, and there is good reason to believe these to be positive charges. A conventional model has become more or less generally accepted [5] in which the channel opens by having the S4 segments move in the extracellular direction in response to depolarization of the cell membrane within which the channel sits. To close the channel, the S4 segments move in the opposite direction in response to repolarization of the membrane. We have suggested that caution is required in interpreting the evidence for this model [6] and have suggested an alternative method for gating [7,8,9] in which protons provide the mobile positive charges, rather than S4 segments as a whole [10,11]. We suggest that what is moving are at least two, and probably three, protons, for each of the four domains. Based on quantum calculations, it is possible to show that at least one proton can be generated from an arginine-glutamate-tyrosine triad of amino acids in the VSD [7,12]. It is known that the H_v_1 channel, which is very similar to the K_v_1.2 VSD in its upper half (approximately) [13,14,15,16], does in fact transmit protons, as does bacteriorhodopsin [17]. The H_v_1 results, albeit with not quite the same interpretation, have been reviewed by deCoursey [18]. All three (K_v_1.2, H_v_1, bacteriorhodopsin) have similar structures for their proton transport sections (in the case of K_v_1.2, for our proposed proton transport section). Helms and Gu found a double proton wire in green fluorescent protein, a case that is an even closer analogue to the proposal presented here [19]. A number of examples of relevant triads of amino acids have been proposed; Chanda and coworkers have explicitly worked out the thermodynamics of an arginine, glutamate, and tyrosine triad in the context of gating [20]. This is the same triad that we found produces a proton by way of tyrosine ionization, as cited above, although Chanda and coworkers did not interpret their result this way. In fact, proton paths in multiple proteins are known, too many to cite here, but we can cite at least some relevant recent works [10,11,21,22,23]. Our proposed mechanism, although it differs from standard channel models, does not involve any new physical or biological principles [24]. Proton paths can also be found both in H_v_1 and in K_v_1 (here, K_v_1.2 specifically) for the sections where the two diverge. In H_v_1, the path proceeds fairly directly to the membrane headgroup region, where the proton can be released [25], while there is a bend in the path in K_v_1. In addition, there are cases of proton transport producing allostery, with, as examples, influenza M2 virus [26], the NHE1 and NHE3 Na+/H+ exchanger transporters [27,28], and other cases [29,30]. It is possible to find what appears to be a proton wire in the intracellular portion of the Kv1 channel that continues from the VSD to the gate, and it this path that we discuss here. Work by Bassetto et al. [31] used alanine substitutions in the linker section, which could easily interrupt a proton wire; the interpretation of that experiment is again *apparently* (a full analysis does not yet seem to be possible) compatible with our model, again if one does not start with the assumption that a mechanical coupling must exist; their interpretation does not contemplate the linker as a possible proton path. Our proposed path includes several amino acids that are known to be important in gating from experimental mutations [24,32]. Proton binding has been found in other channel systems, such as the just cited bacteriorhodopsin, known to transmit protons [33,34]. This is accompanied by proton transport [35], which can be unidirectional [36]. Triads of amino acids, often a salt bridge plus tyrosine, occur in several of these cases; it appears that triads are able to transmit protons effectively. In this work, we see a path composed not only of triads, but in a significant part of longer amino acid sequences, with four or even five amino acids that could function as part of a proton wire. Adding one, or sometimes two, water molecules to bridge these groups makes the paths complete. Two residues can be identified as necessary termini for one proton chain, as we will see in the results. In addition, K_v_1.2 has, at the gate, a group of two asparagines and a serine. Asparagines have already been suggested once as important in coupling the voltage-sensing domain, albeit of a sodium channel, to the gate [37]. The three oxygens (from two asparagines, one serine) in the side chains, given their net local potential, form an energy minimum that would help hold a proton. The sequence, especially the protonated residues, and those that can exchange protonation, is reasonably well conserved over a number of species [38]. The question of proton passage in the H_v_1 channel, involving a salt bridge similar to the one we find to be important, was the subject of a small quantum calculation by Dudev et al. [39]; the results were largely consistent with what we find. The same group also calculated selectivity in the influenza M2 channel, especially with regard to a role for histidine in transmitting a proton, again with results essentially consistent with ours, allowing for the difference in the systems [40]. 

There is a proposed model by Kalstrup, and Blunck for the electromechanical opening of the gate, in which the S4–S5 linker physically pushes the gate closed [41,42]. We note here that the experimental results appear to be compatible with our model, even though they are interpreted by the authors to fit a different, largely mechanical, model. This paper, and several others, use Voltage Clamp Fluorometry (VCF) [43,44] to determine distances between somewhat distant moieties. One such paper from Bezanilla and coworkers showed a very large field, of the order of 10^8^ V m^−1^, locally, near the second arginine in S4, by using a solvatochromic dye to determine the magnitude of the electric field [45]. This has the further implication that the field elsewhere must be small, since dropping 10^8^ V m^−1^ across 7 Å gives a voltage drop of 70 mV, essentially the entire membrane voltage. It appears that one transition, of whatever nature, must drive the entire closing process. It is not clear how, in the standard model, the field might move or change during closing—whether the field moves with the S4, or each arginine passes through the field driving the others, or perhaps some other mechanism. In our model, we would have the start of the proton cascade to which we attribute the gating current, and have shown how an ionization of a tyrosine would occur there [12], thus starting the cascade. The gating current does not appear to be well understood. The magnitude of the gating current was shown by Islas and coworkers to be different in K_v_ 1.2 from the value in the seemingly very similar *Shaker* channel, a surprise, and the interpretation is still not obvious [46]. Islas has gone on to point out the diversity of gating current in K_v_ channels [47]. 

**Figure 1:**
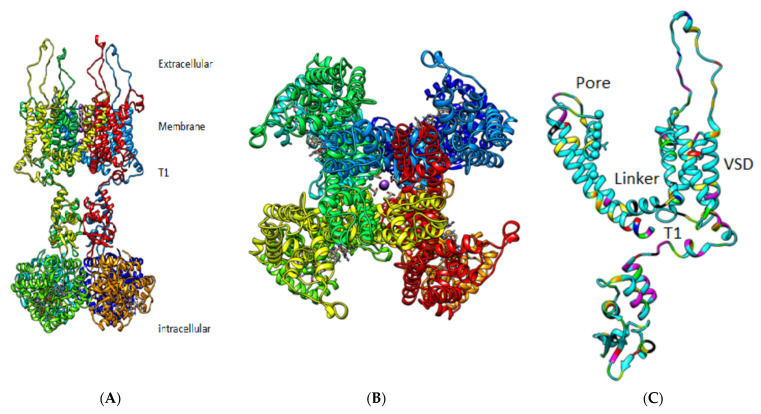
General view of a K_v_1.2 channel from the 3Lut coordinates. (**A**): View from the membrane (side view). Two of four domains are shown, with front and back domains deleted to allow the structure to be seen. (**B**): A 90° rotation of the channel, showing the pore. In neither (**A**) nor (**B**) are individual amino acids shown. The T1 section is somewhat visible in (**A**). Neither (**A**) nor (**B)** make it clear that the linker is not entirely continuous unless the water is included, although water is not shown in these figures. (**C**): A single domain is shown, making the domain crossing clear. Although some individual amino acids are shown with specific colors, it is too difficult in this view to see the relation of the actual amino acids, and again the water is not shown. The region that was calculated is labeled here as linker and T1, although it is hard to see the exact boundary in this type of figure. Figure 2, Figure 3 and Figure 4 show the specific calculations, with individual amino acids and water explicit and clear.

Here we will show how two proton paths can be found that allow protons to connect the VSD to the pore, plus the possibility of crossover between paths. There are a number of papers that report the effects of certain key mutations; these are relevant for our model, as they involve key points in the path from the VSD to pore. One of the two proposed proton paths involves the T1 moiety at the intracellular side of the channel, and it has long been known that the T1 moiety is involved with gating [48,49]; again, alternate interpretations have been suggested, although these alternatives are not always consistent with each other. As far as we can see, the proton path interpretation is consistent with all these data. Furthermore, there are other mutation experiments that point to specific residues as being crucial, including (using the 3Lut numbering) E327 [50] and H418 [24,32]. If either of these is deleted, or in most cases even mutated, the channel fails. Figure 1 gives an overview of the channel.

## 2. Methods

We have carried out quantum calculations on a critical 563-atom section (which includes 28 water molecules) of this path, and can easily see what appears to be a proton wire for linking this to the VSD at one end and to the gate at the other; in fact, two paths can be found, one of which involves the T1 moiety of the intracellular section of the channel, which, based on experiment, is necessary, as noted in the introduction. The quantum optimization (energy minimization) of this portion of the intracellular section of the channel includes much of the linker of the VSD to the pore (the S4–S5 linker, together with a small part of transmembrane segment S6). The optimization was done at the HF/6-31G** level, using Gaussian09 [51]. The calculation starts from the X-ray structure of the open conformation, with the protons added by a normal mode analysis in the 3Lut structure [31,52]. The calculations had the protons on the following pairs of amino acids: (1) K312 and R326; (2) one proton shared by the K312—R326 pair (which we count, for the present, as being on R326), one on E420; (3) E136 (T1) and E420. The two protons were moved initially by hand; they were started in different positions in the three optimizations. In each optimization, the same 33 atoms were frozen, so that there was no possibility of any overall translation of the system. All 33 frozen atoms were C or N atoms that were part of peptide bonds not in either proton path. The optimizations were begun with the water molecules oriented in arbitrary fashion, without any particular order. They were not set up to be hydrogen bonded, nor were any of the side chains of the amino acids. The latter depended in a large part on the X-ray structures; since these did not include water (some oxygens excepted), the hydrogen bonding scheme was not part of the initial setup in any way. The optimizations provided the hydrogen bond networks and proton wires that were found. We determined the distances of the key residues, and the distances to residues (using the X-ray structure) near the VSD that were not included in the calculation (K306, S308, R309), which show that the proton(s) can easily transfer from VSD to the linker, and, at the other end, the distances show transfer by way of E327 or H418. The section that was calculated corresponds to the linker of one VSD, of one domain, to the gate. The total charge on the 563-atom system as calculated is zero whether closed or open; in the calculation, charge is moved, but not added. The energies of the optimized states are determined by B3LYP/6-311G** single point calculation, using NBO [53] on the optimized structures. Charges are determined by the same calculation. 

## 3. Results

**Figure 2:**
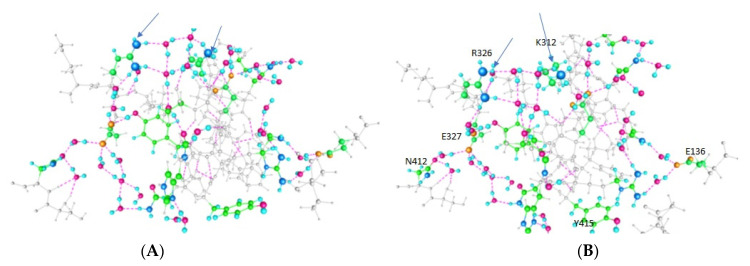
This figure shows two views (system rotated to make it possible to see all the residues clearly) of the results of the optimization with protons on the almost adjacent residues K312 and R326. Key residues are labeled in (**B)**, which is the same as (**A**) but rotated 90°. The energy for this result turns out to be more than 30 k_b_T above the other two cases. The two charged residues separate by more than 2 Å beyond the X-ray value. Both proton paths are easily visible, by following the colored residues, and the connecting (meaning hydrogen bonded) water molecules. The upper path includes K312 and R326, and, in this optimization, ends at N412, from which it has access to the conserved PVPV sequence. The lower path, which includes Y415, continues via H418 to N414, which likewise has access to the PVPV sequence. Other amino acids included in the optimization, but not the proton path, are shown in gray. In addition to the upper and lower paths, there is a crossover that apparently allows protons to switch paths; this is the vertical path in the left-center. The gate is on the left, the VSD on the right and above. Color code: Carbon, green; nitrogen, large blue sphere; hydrogen, small pale blue spheres; oxygen, magenta. The added protons are not visible, but their positions are indicated by the arrows.

**Figure 3:**
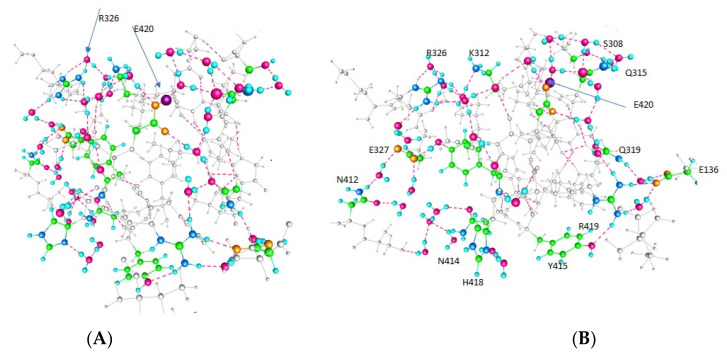
As in Figure 2, the key residues are labeled in (**B**), and the two paths, plus the crossover, can be seen by following the colored residues. The color code is the same, and the arrows again indicate the position of the two mobile protons (in (**A**) R326 is indicated; that residue is always protonated, but is indicated here as well). Rotation from (**A**) to (**B**) is as in Figure 2. This is one of the two low energy configurations, so this arrangement is considered to be part of the actual pathway.

**Figure 4:**
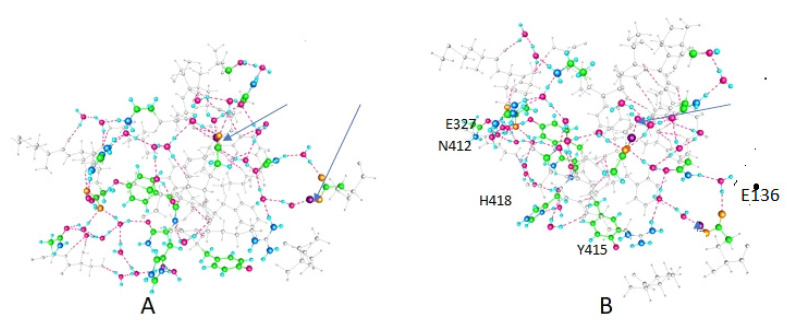
This is the other low energy configuration. All conventions (color code, rotation, arrows for protons) are as in Figure 2 and Figure 3. This case is similar to the X-ray structure. (**A**,**B**) figures correspond to the (**A**,**B**) of Figure 2 and Figure 3; (**B**) contains the labels on specific amino acids.

The principal results are optimized structures, which are shown in Figure 2, Figure 3 and Figure 4. These share certain properties. For one thing, the initially randomly placed water molecules are now all hydrogen bonded, essentially all at normal hydrogen bonding distances. The electronegative atoms in the amino acids also now take part in salt bridges, or are hydrogen bonded either to water or to another electronegative atom on a neighboring amino acid (neighboring in distance, not necessarily near in the peptide sequence). This is true in all three cases. The network percolates through the entire system, which is the key point if protons are to be transmitted. It is sufficient that it is complete. This essentially establishes the key finding, that proton paths, or proton wires, exist through the linker. They use water, and the computation shows that the water will orient so as to complete the proton path. This is a central finding of the computation.

The computations on the 563-atom sections showed that the distances between pairs of atoms in neighboring amino acids could shift by a little more than 2 Å for neighboring residues. Given that certain atoms had to be frozen, it is possible that these distances could shift a bit more in the actual protein. We also need to know the charges on the amino acids to ensure that the path has no unusual barriers. Table 1 gives the charges on the side chains of several of the most important amino acids on the paths. First, in Figure 2, Figure 3 and Figure 4, we show the results of the optimizations. To help understand the figures, Table 1 shows which amino acids are protonated in each figure; each figure corresponds to an optimization. Figure 4 shows how the calculation is oriented with respect to the VSD and gate.

**Figure 5:**
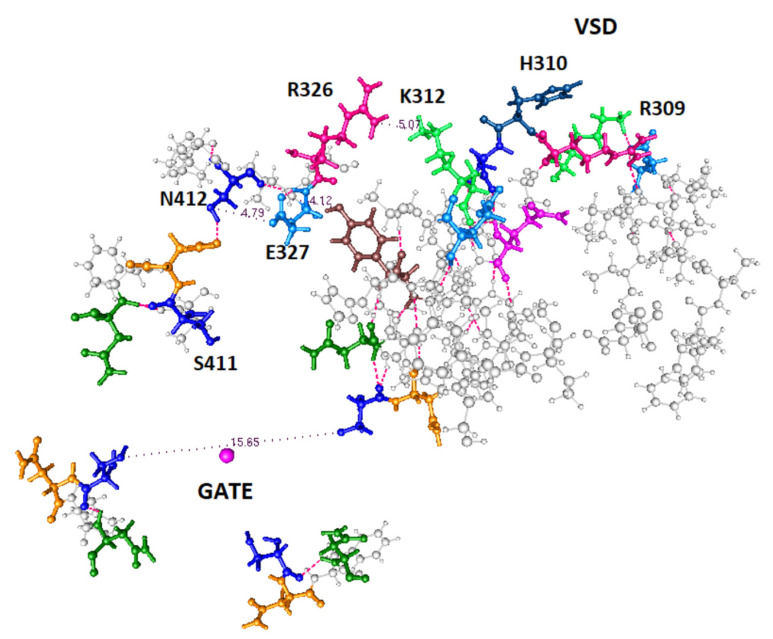
This figure is taken directly from the 3Lut coordinates, and shows the relation of the calculated section, principally the upper path, to the gate and the VSD. Upper path residues are labeled; all else that is included is in gray. R309 and H310 are not part of the calculation, but form the link to the VSD. All four domains at the gate are shown in part, so that the orientation of the linker can be understood.

In Figure 5 we get an overview of the entire system, showing where the gate and VSD are with respect to the remainder of the system.

We can see that the paths appear complete. The actual residues in the upper and lower paths, and the water molecules that connect them, are listed in order in the italicized paths below. Note that the paths can cross at Q315, so a mutation in one path will affect gating, but not kill the channel entirely.

*1: UPPER PATH: S308* *→ W^#^* *→ Q315* *→ W* *→ E420* *→ 4W* *→ K312* *→ W* *→ R326* *→ E327* *→ W* *→ N412*

*2: LOWER PATH: S308* *→ W* *→ Q315* *→ 2W* *→ Q319* *→ (SALT BRIDGE: (W,R419;W,E136)* → Y415** *→ H418* *→ 2W* *→ N414*

*Notes on the paths:*

# Each water (W) has a well-defined position and orientation in the calculation (the position of the water.

Molecules are not arbitrary in the optimized configuration, but are determined by the calculation)

* E136 is from the T1 moiety; each member of this salt bridge is associated with one water.

** the R–E–Y triad appears in several proteins that transmit protons, in their proton paths 

(e.g., bacteriorhodopsin, H_v_1, influenza M2 channel). 

The two proton paths go from VSD to the gate, where N412 and N414 touch the PVPV gate. The water molecules are integral to the paths; in the initial positions from which the calculations begin, waters start in arbitrary positions; when the energy is optimized, the water adopts the positions shown; the calculation shows that they complete the proton paths.

*Completing the path:* Because the section of the linker that is closer to the gate shifts a bit more than 2 Å in going from one of the two low energy states to the next, the distal section of the linker, taking this as a hinge, would shift more. This is significant in that it agrees, to a reasonable approximation, with the VCF results of Kalstrup and Blunck [41]; they report that the S4 and proximal section of the linker remain at the same distance from each other, while the distal section of the linker moves with respect to S4. This is essentially the way the results we calculate would appear, at least qualitatively. The K312—R326 pair turned out to have high energy when both were protonated so that this may be at most a transient state; while the arginine, R326, always remains protonated, the neighboring lysine, K312, does not. In addition, a tyrosine further down, Y315, can be deprotonated. Table 1 shows the protonation states of those residues that are able to exchange protons, with the consequences for conformations illustrated in Figure 2, Figure 3 and Figure 4.

Each case (Figure) has two protonated residues. Figure 4 shows inter-residue distances that correspond fairly closely to the X-ray distances, in particular for K312–R326 distances, while Figure 2, in which both are protonated, shows a much larger distance (>7 Å, compared with 5.5 Å, among other differences). The X-ray distance for Y415–R419 is implausibly small, and the calculated value, while still short, is far more reasonable. The close approach of the K312 and R326 when the proton is on Y415, that is, distant, is interesting; it may be that the one remaining proton is partially shared, giving the bond partial covalent character. This would be a subject for future research. Table 2 gives distances between key residues that help show where certain distances change.

The hydrogen bond network extends, in the calculation, to H418, so that the next transfer, if the channel is closing, would be to the N412 and N414 neighboring pair, as shown in the figures. There is also a fairly close serine, S411, not included in the calculation and thus not shown in the figures, which can connect to these through a water molecule, and help with proton transit to the PVPV section of the gate. Taken together, these should be able to accept a proton and transfer it to the gate. The distances involving S411 (from the X-ray structure) show that the proton should be able to transfer easily to the PVPV sequence at the gate, completing the path at the gate end. S411, together with N412 and N414, forms a triad with oxygens directed so that they could apparently hold a proton electrostatically; however, we have not confirmed this by computation. 

There is a small gap between the earlier computation of the VSD [12] and the present computation of the linker. The residues that were not in either computation, but that do appear to be in the proton path, are K306 and R309. Both of these are part of the S4 set of positive charges, and fit with the path from the VSD. S308, which is in this set of calculations, and can be seen in the upper left of Figure 3B and 4B, includes a hydroxyl that can swivel. The swivel is consistent with the properties of serine, and would allow the proton path to be directed properly; that a serine can rotate in this manner, as part of transmitting a proton, has been established in another system [54]. Again, the distances involved are appropriate for proton transfer.

Thus, at both ends of the calculated linker path, we see that there are residues that are capable of proton transfer, and are placed appropriately for such transfer to occur. If there were mechanical transmission, say of motion of S4, there could just as easily be hydrophobic residues in these locations, and there would be no need to have a linker with what appears to be a complete net for proton transmission. 

Because it appears that there is good conservation of this structure, we consider that it does have significance.

*Charge on the residues:*Table 3 shows charges on the side chains of amino acids that are part of the network that could transmit a proton. “Side chains” include the guanidinium groups of arginine, the carboxyl group of acids (either COO^-^ or COOH, depending on protonation state), the NH_2_ or NH_3_^+^ group for lysine, again depending on whether protonated), and the amide group for asparagine. The charges shown are the sums of the charges on the component atoms.

With the charges, we see how the charges could effect, or at least affect, a sequence of transfers, as well as how the direction of transfer could reverse, depending on the field. 

## 4. Discussion

We can see from the results (figures) that there are two complete paths between the VSD and the gate, plus the possibility of crossover. The energy for two sets of positions of the protons is reasonably close, only about 3 k_B_T apart, and as long as we do not attempt to put two protons too close, the energy can be reasonably expected to stay within roughly the same range. A range of about 15 k_B_T for all positions through which the protons must pass would be acceptable; a value of 15 k_B_T would allow a Q_10_ of roughly 1.6, which is about in the appropriate range. The scatter among measurements requires a certain error bar on this, but for comparison with a path here, this is adequate agreement. The charges and distances are appropriate for the proton transfer, with relatively little driving force. One further calculation of the protonated gate would be useful to fix the final position, and that is left for future work. The current prediction is that this would have a lower energy when polarized (i.e., closed state) by roughly 30 to 40 k_B_T, the amount which would be available if 70 mV were applied to 10 to 12 charges (four domains, up to three charges each); with four domains, this is approximately the energy available from the work that can be done by membrane polarization, to pull the charges down to close the channel. This makes the open state more thermodynamically stable with the membrane depolarized, and requires that work be done to close the channel. When the membrane is depolarized, the charges should move up the VSD to allow the gate to open, and the motion of these charges is the gating current. 

The calculation shows the position of several key residues. On the lower path, these include E136 of the T1 residue, in addition to several other residues that are part of the path, including Y415 and R419. Mutations test the importance of residues for the path, or for any mechanism. Not all residues have been mutated, but several have. For our purposes, the most important are E327, H418, R326, and the effect of the T1 moiety on gating. All these appear as key residues in our paths. 

There is a special case worth separate discussion. On the upper path, the K312-R326 pair cannot both be simultaneously protonated; double protonation produces too high an energy to be in any plausible path. On the other hand, they appear, when sharing a single proton, to form a bound pair at a short distance from each other, and are therefore likely to be a key part of the upper path. A proton passing this pair would cause a significant shift in the distal part of the linker; while the two come only 2.2 Å closer, compared to X-ray or the third case (Figure 4), this also produces a hinge motion that, it appears, would cause a further shift closer to the gate. 

In addition, we are able to point to the connections at either end of the paths. There are residues at the correct distance from the ends of the calculated sections to allow the proton to continue to the VSD at one end (K306, R309), and to the gate at the other (S411, connected to N412, N414 from the paths, and leading to the PVPV section at the gate end). We commented, in the results section, that the presence of residues that would complete the proton paths at both ends would seem odd if there were a mechanism that did not involve proton transfers.

In order to have the path complete, we have in several key locations a serine or a threonine; the hydroxyl can swing through more than 90°, thus redirecting a passing proton. It is interesting to note that the serine or threonine always appears to be placed at exactly the position needed to continue the path, especially where redirection is required.

We can, in other words, see how the protons proceed between the VSD and the gate, in either direction. There is no indication that the S4–S5 linker shifts on gating or closing the channel, so essentially the same path should apply to proton transfer in both directions. However, this does not rule out hysteresis, as there are two paths, and a connection between them; very extensive calculations would be needed to show the detailed energy paths (each step in proton positions, with accompanying energy, and transition states between these) in each direction. In particular, it may provide a lower energy, or activation energy, path if the crossing path transfer is included in one direction but not the other, which could happen if the activation energy along the path caused the kinetics to force a change in direction. Therefore, the lack of movement of the linker does not rule out hysteresis. 

The calculation that we have done produces a result that is consistent with the hypothesis that protons are responsible for gating, as discussed in the introduction. In one sense, we could have simply looked at the X-ray structure, noted the distances and the presence of residues that are appropriate for transmitting protons, and immediately stated that protons could be transmitted. However, the calculation shows how the water molecules line up, and this is important to ensure that there is not some additional factor that we might have missed. Instead, the water lined up as necessary for the proton wire, completing the network of hydrogen bonds; this could not have been concluded *a priori*. This calculation is not quite sufficient to totally prove the proton gating model, but it does represent strong support for this model. With this calculation, and the earlier VSD calculation, we have almost the entire proton paths needed for a complete gating mechanism; only the gate itself, and perhaps some overlapping with connecting sections, remains, and the necessary paths are apparent already from the work done so far, and from the X-ray structures. 

## 5. Conclusions

(1) Two proton paths between the VSD and the gate can be clearly defined. A crossover path between them also exists.
(2) We have ruled out a conformation in which K312 and R326 are simultaneously protonated, while the configurations with one or zero protons on these two residues have roughly equal lower energy.
(3) There are appropriate, proton transmitting, residues at each end of the computed path; it is possible to see how the proton connects both to the VSD at one end, and to the gate at the other.
(4) The paths appear to include local energy minima for protons, at which the protons are temporarily immobile. For example, groups of three amino acids which have fairly close oxygens would very likely form such a minimum; calculations on the details are ongoing. In a proton cascade, a proton coming from a higher energy location would displace the proton in such a local minimum, pushing it forward along the proton path.
(5) The charges and the bonding have been determined for all key residues with three proton conformations, two of which are considered to actually be part of the paths.

## Figures and Tables

**Table 1:** Protonation States of Those Residues that can Exchange Protons.

Amino Acid	Figure 2	Figure 3	Figure 4
E420	--	+	+
Y415	+	+	--
K312	+	--	--
E136 (In T1)	--	--	+

where + indicates that the residue is protonated, and—indicates that it is not protonated.

**Table 2:** Comparison of Certain Distances (Å) with X-ray Structure Distances *.

Amino Acids	Figure 2	Figure 3	Figure 4	X-ray
R326–K312	7.11	3.35	5.55	5.07
E420–Q315	4.67	4.66	4.58	3.46
Y415–R419	3.14	3.20	2.60	2.04
R419–E136	4.09	3.00	4.89	5.08

* Corresponding specific N and O atoms in each pair.

**Table 3:** Charges on side chains of important amino acids.

Amino Acid	Figure 2	Figure 3	Figure 4
E327	−0.782	−0.785	−0.798
R326	0.845	0.828	0.808
K312	0.594	−0.172	−0.143
E420	−0.791	−0.598	−0.538
Q315	0.026	0.042	0.024
R419	0.857	0.853	0.783
E136	−0.772	−0.762	0.009

## Data Availability

The coordinates of the atoms in the optimized calculations are available on request from the authors (mgreen@ccny.cuny.edu).
